# Fucoidan–Vegetable Oil Emulsion Applied to Myosin of Silver Carp: Effect on Protein Conformation and Heat-Induced Gel Properties

**DOI:** 10.3390/foods13203220

**Published:** 2024-10-10

**Authors:** Wei Wang, Lijuan Yan, Shumin Yi

**Affiliations:** National & Local Joint Engineering Research Center of Storage, Processing and Safety Control Technology for Fresh Agricultural and Aquatic Products, National R&D Branch Center of Surimi and Surimi Products Processing, College of Food Science and Engineering, Bohai University, Jinzhou 121013, China; wwll812002@163.com (W.W.); 2021015033@qymail.bhu.edu.cn (L.Y.)

**Keywords:** myosin, fucoidan, vegetable oil, protein conformation, gel properties

## Abstract

How to improve the gel properties of protein has become a research focus in the field of seafood processing. In this paper, a fucoidan (FU)–vegetable oil emulsion was prepared, and the mechanism behind the effect of emulsion on protein conformation and the heat-induced gel properties was studied. The results revealed that the FU–vegetable oil complex caused the aggregation and cross-linking of myosin, as well as increased the surface hydrophobicity and total sulfhydryl content of myosin. In addition, the addition of the compound (0.3% FU and 1% vegetable oil) significantly improved the gel strength, hardness, chewiness, and water-holding capacity of the myosin gel (*p* < 0.05). In particular, when the addition of camellia oil was 1%, the gel strength, hardness, chewiness, and water-holding capacity had the highest values of 612.47 g.mm, 406.80 g, 252.75 g, and 53.56%, respectively. Simultaneously, the emulsion (0.3% FU-1% vegetable oil) enhanced the hydrogen bonds and hydrophobic interaction of the myosin gels. The image of the microstructure showed that the emulsion with 0.3% FU-1% vegetable oil improved the formation of the stable three-dimensional network structure. In summary, the FU–vegetable oil complex can promote unfolding of the protein structure and improve the gel properties of myosin, thus providing a theoretical basis for the development of functional surimi products.

## 1. Introduction

Usually, surimi is obtained by undergoing chopping and rinsing from freshwater fish. The rinsing process removes large amounts of lipids and water-soluble proteins from the surimi [1]. However, many studies have shown that lipids can not only improve the nutritional content but also improve the quality of surimi products [2,3]. To tackle this problem, lipids are often added as improvers, colorants, and processing aids to improve the texture, whiteness, and nutritional properties of surimi gel. Liu et al. [4] found that 30% pork fat showed the best gel properties. Although the addition of animal fat can improve the gel properties of surimi products, excessive intake of saturated fatty acids and cholesterol is harmful to human health. Thus, different vegetable oils such as peanut oil, rapeseed oil, and corn oil are added to surimi products to modify their gelling properties [5].

Vegetable oil is rich in unsaturated fatty acids, various vitamins, and other functional components, which can appropriately enrich the nutrients of surimi products [6]. Camellia oil and soybean oil are good sources of nutrition which contain a lot of unsaturated fat acids; for example, camellia oil contains linoleic acid (10.06%), oleic acid (77.81), and linoceric acid (8.12%) and soybean oil contains linoleic acid (50.3%), oleic acid (23.7%), and palmitic acid (10.9%) [7,8]. Studies suggested that the direct addition of vegetable oil to surimi products has negative effects on their texture and gel. However, pre-emulsified vegetable oil is beneficial to the formation of a denser protein–network structure. Youssef et al. [9] found that the microstructure of surimi could be significantly improved by adding pre-emulsified lipids. Sadly, under high temperatures and during refrigeration, and cooking processes, lipid oxidation will still lead to a quality decrease in surimi products. In order to solve this problem, finding an emulsifier that can enhance gelation and protect against oil oxidation is really important.

Fucoidan (FU), a kind of natural water-soluble polysaccharide, is composed of sulfated fucose, mannose, arabinose, glucuronic acid, and other monosaccharides, which has biological functions such as antioxidant activity, immune regulation, and anti-tumor and antibacterial effects [10,11,12]. Furthermore, due to its hydrocolloid properties, FU can be used as gelling agents, stabilizers, thickeners, and emulsifiers in the food industry [13]. Due to its unique nutritional value, good gelling characteristics, and strong water-holding and biological activities, FU can be used as a emulsifier-forming emulsion to improve the gel properties of surimi products.

As we all know, myosin plays a key role in the quality of surimi products. However, few studies have reported on the effect of combining FU with vegetable oils on the physicochemical properties of myosin. Thus, this study explored the mechanism behind the interaction among FU, vegetable oil (camellia oil and soybean oil), and myosin on the conformation and gel properties of myosin, aiming to provide a theoretical reference for the development of surimi products.

## 2. Materials and Methods

### 2.1. Materials

FU (purity > 98%) was purchased from Guangzhou Six Flavors Biotechnology Co., Ltd. (Guangzhou, China), and camellia oil and soybean oil were acquired from Fujian Shenlang Oil Tea Co. (Sanming, China) and Kerry Food Oil Tianjin Co., Ltd. (Tianjin, China), respectively. Silver carp with an average weight of (2.0 ± 0.5) kg was obtained from the seafood market in Jinzhou (Liaoning, China). All analytical reagents were of analytical grade and used directly without further treatment.

### 2.2. Preparation of the FU–Vegetable Oil–Myosin Emulsion

Myosin was extracted according to the method described by Wang et al. [14] with minor modifications. The myosin concentration was adjusted to 10 mg/mL, and then, 0.3% FU (based on the results of a pre-experiment) and vegetable oil (1–3%, camellia oil and soybean oil) were added. Finally, the mixed solution was homogenized at 12,000 rpm for 1 min.

### 2.3. Preparation of the Myosin Gel

FU-based emulsions were prepared using a high-pressure homogenizer. Vegetable oil (1%, camellia oil or soybean oil) was mixed with FU (0.3%), sheared at 12,000 rpm for 5 min, and then homogenized at 40 MPa twice.

The preparation of the myosin gel referred to the method of Wang et al. [14]. Myosin was adjusted a concentration to 40 mg/mL. Thereafter, FU (0.3%) and vegetable oil (1–3%) were added to the myosin solutions. A high-shear homogenizer was applied to homogenize the mixtures. After 1 h of equilibrium at 4 °C, the samples were placed into a water bath for two-step heating (40 °C for 30 min and 90 °C for 30 min). The samples were refrigerated at 4 °C for 24 h to obtain the composite gels.

### 2.4. SDS-PAGE

The method followed for sodium dodecyl sulphate-polyacrylamide gel electrophoresis (SDS-PAGE) referred to Yi et al. [15] with minor modifications. In this experiment, 10% of the separating gel and 5% of the stacking gel were used. The electrophoresis was performed in a vertical unit (Mini PROTEAN Tetra, Bio-Rad, American). After completion of the electrophoresis, the gel was stained with a protein fast staining solution. Scanning and analysis were performed using a GS-800 image scanner (Bio-Rad, Hercules, CA, USA).

### 2.5. Measurement of Determination of Turbidity

The turbidity was determined according to the method described by Mi et al. [16] with some modification. The sample solution was measured at 350 nm with a spectrophotometer (UV-2550, Shimadzu Instrument Co., Ltd., Kyoto, Japan).

### 2.6. Measurement of Particle Size

A particle size of 0.1 mg/mL of myosin solution was determined with a Nano Brook 90 Plus particle size analyzer (Brookhaven Instruments Corporation, Holtsville, NY, USA) according to a relative method [17].

### 2.7. Measurement of UV Absorption Spectroscopy

The UV absorption spectroscopy was carried out according to the method of Yi et al. [18]. The ultraviolet–visible (UV) spectra of the sample (0.2 mg/mL) were investigated using a spectrophotometer ranging from 240 nm to 320 nm.

### 2.8. Measurement of Fluorescence Spectroscopy

The intrinsic fluorescence spectrum of the myosin (0.4 mg/mL) was measured with a 970 CRT fluorescence spectrophotometer (Shanghai Precision Scientific Instrument Co., Ltd., Shanghai, China) according to the method of Zhou et al. [19].

### 2.9. Measurement of Total Sulfhydryl (SH) Content

The total SH content was measured as described by Wei et al. [20] with minor modification.

### 2.10. Measurement of Surface Hydrophobicity

The surface hydrophobicity was determined as described by Cheng et al. [21]. The myosin–FU or myosin–FU–vegetable oil mixed solution was diluted to 0.2, 0.4, 0.6, 0.8, or 1.0 mg/mL and mixed with 8-Anilino-1-naphthalenesulfonic acid for 10 min.

### 2.11. Measurement of Dynamic Rheology

The dynamic rheology was measured following the method of Cao et al. [22] with minor modification. The strain and frequency were 2% and 0.1 Hz, respectively.

### 2.12. Measurement of Gel Strength and Texture

The gel strength and texture of the myosin gels were determined with a texture analyzer (TA.XT Plus, Stable Micro System, Godalming, Surrey, UK). The gel strength was analyzed with a cylindrical probe (P/5S), and the TPA of the composite gel was analyzed with a cylindrical probe (P/50).

### 2.13. Measurement of Whiteness

The whiteness of the gel sample was measured using a color difference meter (CR-400 Konica, Konica Minolta Holdings, Tokyo, Japan). Each group of samples was tested eight times in parallel. The whiteness was calculated as follows:

$$Whiteness=100-\sqrt{{\left(100-L^{*}\right)}^{2}+{\left(a^{*}\right)}^{2}+{{(b}^{*})}^{2}}$$

Here, L*, a*, and b* represent the brightness, degree of red or green, and degree of yellow or blue, respectively.

### 2.14. Measurement of Water-Holding Capacity (WHC)

Water-holding capacity (WHC) is expressed as the percentage of gel weight after centrifugation (3000× *g*, 10 min, 4 °C) versus gel weight before centrifugation.

### 2.15. Measurement of Chemical Forces

The chemical forces in the myosin gels were determined with the method of Wang et al. [14]: 2 g of the myosin gel were added to 10 mL of 0.05 M NaCl (S1), 0.6 M NaCl (S2), 0.6 M NaCl + 1.5 M urea (S3), and 0.6 M NaCl + 8 M urea (S4). The resulting solutions were mixed and homogenized; then, the mixture was kept at 4 °C and fully dissolved for 1 h, followed by centrifugation at 10,000× *g* for 15 min. The contents were calculated according to the method described by Gao et al. [23].

### 2.16. Measurement of Raman Spectroscopy

The samples were cut into small pieces and put evenly onto slides. Raman spectra (LabRAM HR Evolution, HORIBA Scientific, Paris, France) were scanned in the range from 400 to 2000 cm^−1^ using a 532 nm laser.

### 2.17. Measurement of Confocal Laser Scanning Microscopy (CLSM)

The vegetable oil and protein were stained with 0.1% Nile Blue (labeled protein), 0.1% Nile Red (labeled oil), and 0.1% Calcofluor White (labeled polysaccharide) according to the method described by Feng et al. [24] and Zhang et al. [25] with minor modifications.

### 2.18. Measurement of Optical Microscopy

The samples were cut into cubes and frozen. The microstructure of the gels was assessed using HE staining.

### 2.19. Statistical Analysis

The data were obtained and plotted with SPSS 19.0 and Origin 9.0. All data were expressed as the mean ± SD. The significant difference was measured at the *p* < 0.05 level for the one-way ANOVA and bivariate correlation analysis.

## 3. Results and Discussion

### 3.1. Effect of the Emulsion on Morphology and Conformation of Myosin

#### 3.1.1. Protein Patterns

**Figure 1:**
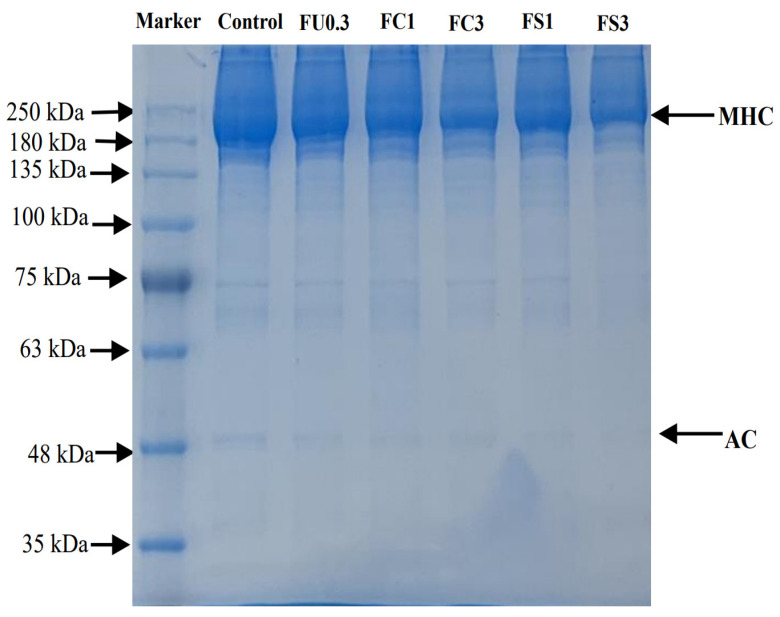
SDS–PAGE profile of myosin. Note: control: myosin gel; FU0.3: myosin-0.3%FU; FC1: myosin-0.3%FU-1% camellia oil; FC3: myosin-0.3%FU-3%camellia oil; FS1: myosin-0.3%FU-1% soybean oil; FS3: myosin–0.3%FU–3%soybean oil.

SDS-PAGE can explain the effect of the FU and FU–vegetable oil complex on protein cross-linking, aggregation, and degradation at the molecular level [26]. Figure 1 shows a thick band appearing at 220 kDa, while no obvious bands appeared at other molecular weights. This result suggests that the purity of the myosin reached the experimental requirements. The band intensity of the MHC was weakened after the addition of FU or the FU–vegetable oil emulsion in contrast to the control group. In addition, the intensity of the bands of the MHC decreased with an increase in vegetable oil. This result suggests that FU and vegetable oil promote cross-linking of the MHC to form larger-molecular-weight peptide chains. From the results, it can be assumed that FU or vegetable oil could cross-link proteins via hydrogen bonds and hydrophobic interactions, thus causing a protein conformation to unfold [27].

#### 3.1.2. Turbidity

**Figure 2:**
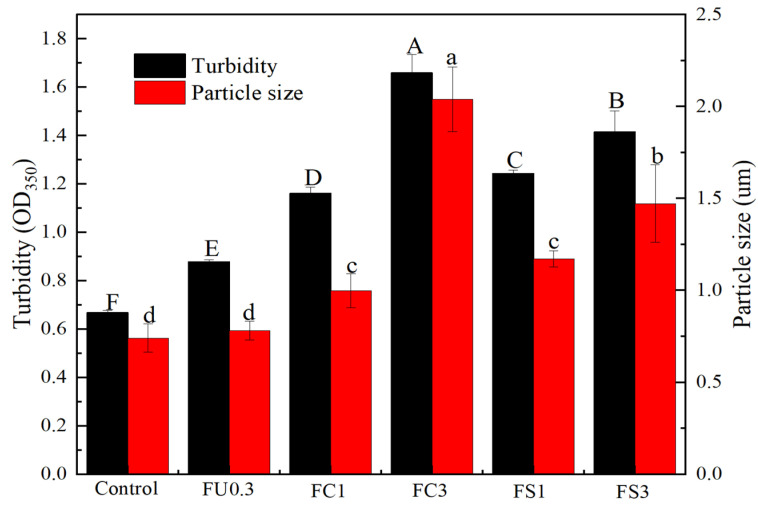
Changes in turbidity and particle size of myosin. Note: control: myosin gel; FU0.3: myosin-0.3%FU; FC1: myosin-0.3%FU-1% camellia oil; FC3: myosin-0.3%FU-3%camellia oil; FS1: myosin-0.3%FU-1% soybean oil; FS3: myosin-0.3%FU-3%soybean oil. Different letters (a–d, A–F) in the same indicator show significant differences at *p* < 0.05.

Turbidity can be used to reflect the number and size of suspended particles in the solution [28]. Figure 2 shows that the diameter of the myosin was continuously increased with the increase in FU-vegetable oil emulsion. The increase in turbidity confirmed that interactions occurred between the myosin and FU or vegetable oil molecules, leading to destruction of the hydration layer on the myosin surface due to competitive hydration [29]. Similarly, Teng et al. [30] reported an increase in the turbidity of the myofibrillar protein with the addition of blackberry crude polysaccharides. The study of Liu et al. [31] showed that the content of konjac polysaccharides could increase the turbidity of myofibrillar proteins. However, different oils have different influences on turbidity, which mainly depends on the ratio of protein and vegetable oil concentration [32].

#### 3.1.3. Particle Size

Particle size can reflect the degree of dissociation or monomer aggregation of myosin [33]. As exhibited in Figure 2, the particle size of myosin increased with the concentration of FU–vegetable oil emulsions. The vegetable oil attached to the surface of the myosin, which led to the aggregation of myosin [34]. The phospholipid molecules in the liposomes introduced additional negatively charged groups, which enhanced the electrostatic repulsion among myosin molecules [35]. Usually, the enhancement of electrostatic repulsions reduces colloidal particle aggregation, resulting in the formation of smaller particles [36]. When the ratio of lipids continues to increase, myosin will aggregate and generate larger particles. The results indicate that the combination of lipids and myosin mainly contributes to hydrogen bonds and hydrophobic interactions, not electrostatic forces.

#### 3.1.4. UV Absorption

**Figure 3:**
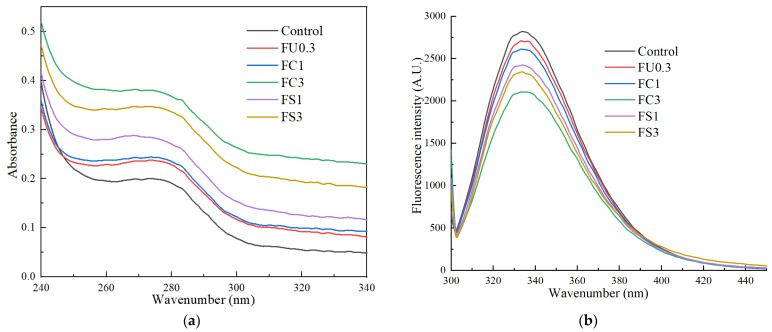
Changes in UV absorption (**a**) and intrinsic fluorescence spectrum (**b**) of myosin. Note: control: myosin gel; FU0.3: myosin-0.3%FU; FC1: myosin-0.3%FU-1% camellia oil; FC3: myosin-0.3%FU-3%camellia oil; FS1: myosin-0.3%FU-1% soybean oil; FS3: myosin-0.3%FU-3%soybean oil.

The main chromogenic groups are tryptophan, tyrosine, and phenylalanine residues of proteins ranging from 200 nm to 380 nm [37]. As displayed in Figure 3a, an enhancement in the peak intensity (280 nm) of the myosin–FU solution was observed compared with the control group, which might be due to the exposure of aromatic amino acids buried in the inside of the protein. The absorption value of the sample (FU–vegetable oil–myosin) at 280 nm was enhanced with the increase in vegetable oil. The reason might be that the amino acid microenvironment or protein conformation changed when the protein combined with the FU–vegetable oil emulsion.

#### 3.1.5. Intrinsic Fluorescence Spectrum

Intrinsic fluorescence spectra are frequently applied to reflect the conformation of proteins since myosin contains important amino acid residues, such as tryptophan (Trp), tyrosine (Tyr), and phenylalanine (Phe), showing fluorescence properties [38]. As illustrated in Figure 3b, the strongest emission peak of myosin appeared near 336 nm. The endogenous fluorescence intensity of tryptophan (Trp) and tyrosine (Tyr) decreased with increasing FU–vegetable oil emulsion. In addition, the addition of the FU–vegetable oil emulsion redshifted the strongest emission peaks of myosin slightly. FU and vegetable oil accelerated the unfolding of the myosin structure, causing exposure of the tryptophan residues, thus reducing the fluorescence intensity [28,39].

#### 3.1.6. Total Sulfhydryl (SH)

**Figure 4:**
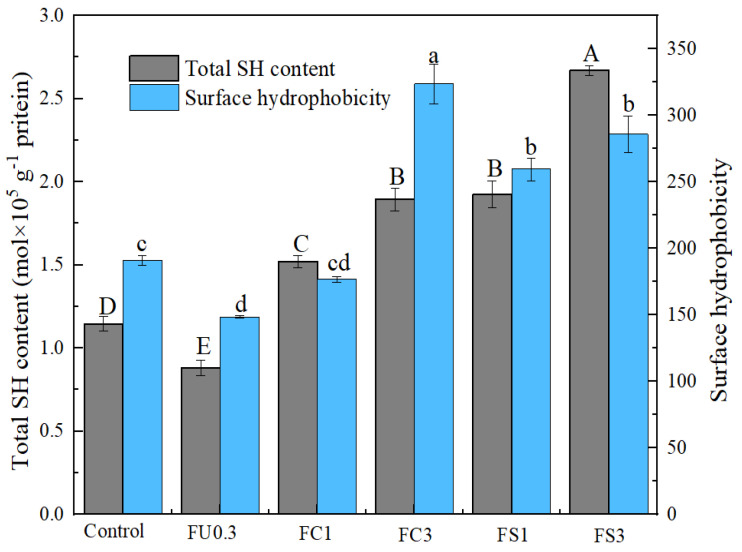
Changes in total SH content and surface hydrophobicity of myosin after adding the FU-vegetable oil complex. Note: control: myosin gel; FU0.3: myosin0.3%FU; FC1: myosin-0.3%FU-1% camellia oil; FC3: myosin-0.3%FU-3% camellia oil; FS1: myosin-0.3%FU-1% soybean oil; FS3: myosin-0.3%FU-3% soybean oil. Different letters (a–d, A–E) in the same indicator show significant differences at *p* < 0.05.

Sulfhydryl groups have a great effect in maintaining the stability of the spatial structure of myosin [40]. Figure 4 shows that the content of total sulfhydryl in the FU 0.3 group decreased compared with that of the control group. This was ascribed to the abundant hydroxyl groups of FU, which could attack the sulfhydryl groups of myosin to generate disulfide bonds [41]. However, the total sulfhydryl content of myosin increased with the addition of the FU–vegetable oil emulsion. This may be attributed to the fact that the myosin structure unfolded, thus transforming the disulfide bond into a sulfhydryl group [42].

#### 3.1.7. Surface Hydrophobicity

The surface hydrophobicity can be used to reflect the conformational changes in the proteins [43]. Figure 4 depicts the notable surface hydrophobicity decrease when 0.3% FU was added. The positive charge on the surface of the myosin and the negative charge on the surface of the FU attracted each other electrostatically, thus forming a myosin–FU electrostatic complex. Studies have implied that FU is rich in hydrophilic hydroxyl groups (-OH), increasing the hydrophilicity of the myosin–FU complex [44]. However, compared with the control group, the surface hydrophobicity of the myosin complex increased with the addition of FU–vegetable oil (Figure 4). This reason may be that the hydrophobic long chain of the fat facilitated exposure of the protein hydrophobic groups [45]. Similarly, Zhou et al. [38] found that the surface hydrophobicity of myofibrillar proteins significantly increased within lower fat additions.

#### 3.1.8. Dynamic Rheometry

**Figure 5:**
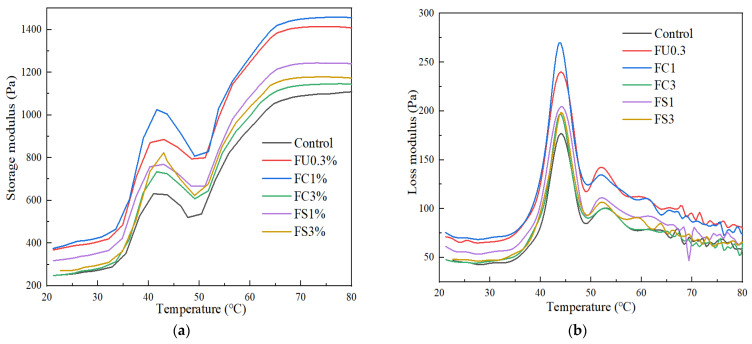
Changes in storage modulus (G’) (**a**) and loss modulus (G″) (**b**) of myosin. Note: control: myosin gel; FU0.3: myosin-0.3%FU; FC1: myosin-0.3%FU-1% camellia oil; FC3: myosin-0.3%FU-3%camellia oil; FS1: myosin-0.3%FU-1% soybean oil; FS3: myosin-0.3%FU-3%soybean oil.

The rheology properties can be used to reflect the denaturation, aggregation, and molecular conformation of proteins [46]. The gelation process of myosin is generally divided into three stages: gel formation, weakening, and enhancement [47,48]. As shown in Figure 5, the increase in G’ value from 40 °C to 20 °C was attributed to the cross-links of the myosin head by hydrogen bonds [49]. The decrease in G’ values from 40 °C to 50 °C was due to the dissociation of the light chain of myosin, causing an enhancement in the molecular mobility, thus resulting in the breakage of the low-temperature gel network structure [26]. The G’ value increased rapidly when a temperature above 50 °C. This is presumably due to the cross-links of the protein aggregates [50].

As exhibited in Figure 5, the samples of FC1 or FS1 showed higher G’ and G″ values than FU0.3, which indicates that vegetable oil and FU had a synergistic effect on the rheological properties and thermal stability of the myosin gels. Generally, FU can induce the unfolding of myosin molecules, causing the exposure of reaction terminals on the protein surface and enhancement of the intermolecular interaction force [51]. Moreover, the types and concentration of vegetable oil also affect the stability of the myosin gel. Zhou et al. [1] found that the addition of camellia seed oil could increase the G’ and G″ values of silver carp myofibrillar protein and was affected by the addition of excess vegetable oil concentration, which was not conducive to the interactions between protein molecules. Shi et al. [5] found that vegetable oil could form large oil droplets and affect the protein–protein interaction, thus leading to a decrease in the G’ and G″ values.

### 3.2. Effect of the Emulsion on the Gel Properties of Myosin

#### 3.2.1. Gel Strength of the Myosin Gel

**Figure 6:**
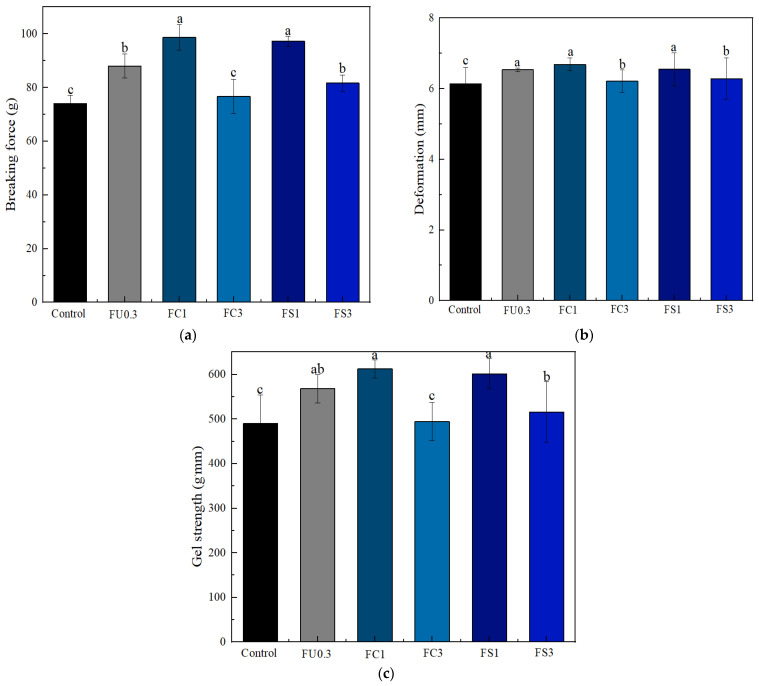
Changes in breaking force (**a**), deformation distance of the gel (**b**), and gel strength (**c**) of myosin gel. Note: control: myosin gel; FU0.3: myosin-0.3%FU; FC1: myosin-0.3%FU-1% camellia oil; FC3: myosin-0.3%FU-3%camellia oil; FS1: myosin-0.3%FU-1% soybean oil; FS3: myosin-0.3%FU-3%soybean oil. Different lowercase letters (a–c) in the same indicator show significant differences at *p* < 0.05.

Figure 6 displays the results of the gel strength of myosin for different samples. The gel strength of the 0.3% FU–myosin gel improved significantly (*p* < 0.05) compared with that of the control group. This phenomenon may be attributed to the interactions between FU and myosin due to the formation of hydrogen bonds and electrostatic forces, thereby enhancing the gel strength [51]. In addition, FU can act as a filler evenly dispersed throughout the gel network [52]. The addition of FC1 or FS1 further increased the gel strength of the myosin gel, which was probably because the FU–vegetable oil emulsion formed small fat globules and filled the pores of the gel protein network [45]. However, the breaking force decreased when the concentration of vegetable oil (FC3 and FS3) increased. Zhang et al. [53] found that the gel strength of surimi gel decreased when the content of the high-internal-phase emulsion increased. The reason may be that the addition of 3% vegetable oil led to an increase in the oil droplet diameter, thus affecting the interaction between the myosin molecules and destroying the three-dimensional network structure.

#### 3.2.2. TPA of Myosin Gel

Texture, an important quality parameter, can be used to reflect the texture of food. As shown in Table 1, FU and the FU–vegetable oil emulsion significantly affected the protein gel (*p* < 0.05). When adding 0.3% FU to myosin gels, the hardness of FU 0.3 (387.41 g) was higher than that of the control group (343.88 g). The hardnesses of FC1 and FS1 were 1.18 and 1.16 times that of the control group, respectively. This was because smaller oil droplets filled the protein continuous phase, promoting the cross-linking of myosin and improving the hardness of the gel. However, when the concentration of vegetable oil kept increasing, the texture of the myosin gel deteriorated, thus causing a decrease in hardness in the FC3 and FS3 groups. This was because the excess lipids affected the formation of the network structure [54]. Shi et al. [5] revealed that excessive vegetable oil affected the interactions of the proteins, thereby hindering the formation of the gel network.

#### 3.2.3. Whiteness and WHC of the Myosin Gel

The whiteness of the different samples is exhibited in Table 2. The result indicates that FU affected the light scattering, causing a decrease in the whiteness of the myosin gels. However, the addition of camellia oil or soybean oil–FU improved the whiteness of the myosin gel. This may be because the oil droplets in the surface of myosin enhanced the astigmatism effect of the gel [55].

WHC reflects the capacity of the gel to trap water. The dense and uniform gel network can reduce water loss and improve WHC [56]. Table 2 indicates that the WHC of FU–myosin gels had better WHC compared with the control group. When both 0.3% FU and 1% vegetable oil were added, the myosin gel had a better water-holding capacity (Table 2). FU is highly hydrophilic and has abundant -OH groups, which can strongly bind water molecules via ionic and hydrogen bonds. The experiment group (0.3% FU-1% vegetable oil) also showed good water-holding capacity, which may be because the fine oil droplets filled the cavities in the protein matrix as a filling material, inducing the formation of a more ordered and dense spatial network (protein–vegetable FU–oil) structure. Specially, FC1 exhibited better particulate filling effects on the composite gels compared with FS1, which effectively reduced the moisture channels and held more water. However, the WHC of the component weakened along with an increase in vegetable oil, which contributed to the formation of larger oil droplets [53].

#### 3.2.4. Chemical Interactions of Myosin Gel

**Figure 7:**
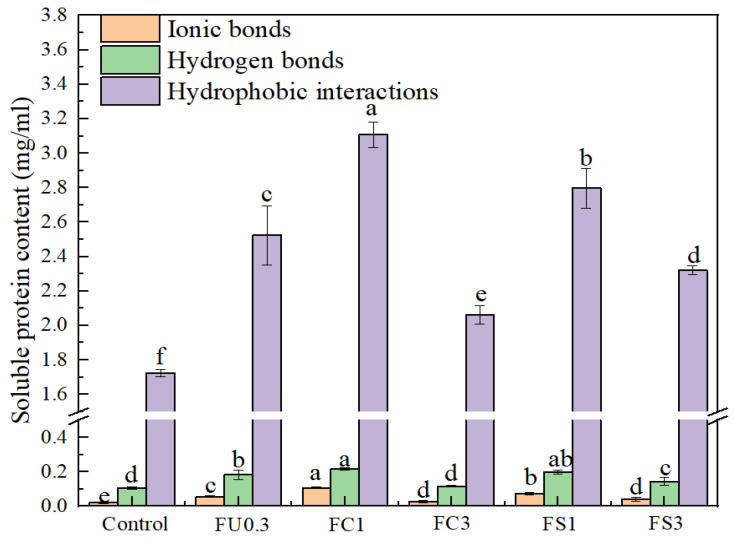
Changes in the chemical interactions of the myosin gel. Note: control: myosin gel; FU0.3: myosin-0.3%FU; FC1: myosin-0.3%FU-1% camellia oil; FC3: myosin-0.3%FU-3%camellia oil; FS1: myosin-0.3%FU-1% soybean oil; FS3: myosin-0.3%FU-3%soybean oil. Different lowercase letters (a–f) in the same indicators show significant differences at *p* < 0.05.

Non-covalent bonds (ionic bonds, hydrogen bonds, and hydrophobic interactions) play a useful role in the thermally induced gelation process. The content of hydrophobic interactions was significantly higher than those of hydrogen and ionic bonds in all groups (*p* < 0.05) (Figure 7). These findings suggested that hydrophobic interaction took a critical role in the formation of the myosin gel, which was consistent with the result of Wang et al. [54]. It can be clearly seen from Figure 7 that the hydrogen bonding and hydrophobic interaction contents of FC1 and FS1 significantly increased (*p* < 0.05), which demonstrated that the FU–vegetable oil complex promoted cross-linking among the protein molecules. However, the contents of the hydrophobic bonds decreased when the concentration of vegetable oil added was 3%. This may be because the particle size of the oil droplet increased the distance between molecules, weakening the interactions between the proteins [42].

#### 3.2.5. Raman Spectroscopy of the Myosin Gel

**Figure 8:**
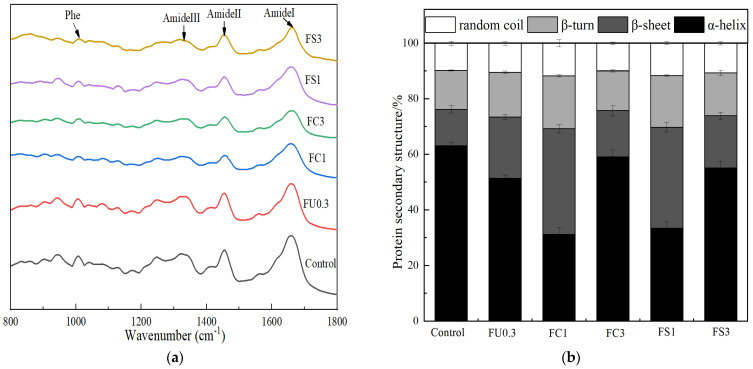
Changes in Raman spectrum (**a**) and protein secondary structure (**b**) of the myosin gel. Note: control: myosin gel; FU0.3: myosin-0.3%FU; FC1: myosin-0.3%FU-1% camellia oil; FC3: myosin-0.3%FU-3%camellia oil; FS1: myosin-0.3%FU-1% soybean oil; FS3: myosin-0.3%FU-3%soybean oil.

Figure 8a indicated that various samples provided similar information at 800 to 1800 cm^−1^. The band at 1600–1700 cm^−1^ (amide I band) represents the C=O stretching vibration of the amide group, the in-plane bending of N–H, and stretching of the C–N bonds [57].

The amide I band commonly provides information about the protein’s secondary structure. Figure 8b shows that the content of α-helix decreased and the content of β-sheet increased with the addition of FU and the FU–vegetable oil emulsion. The α-helix of FC1 had the lowest value (31.17%), and β-sheet had the highest value (38.08%). Generally, the decrease in α-helix indicates an increase in the degree of unfolding in myosin during the gelation process. The increase in β-structures indicated the formation of the gel [58]. The exposure of the hydrophobic groups increased the protein–protein and protein–lipid interactions, resulting in an increase in β-sheets [59]. This result indicates that FU and vegetable oil have a synergistic effect in forming the myosin gel.

#### 3.2.6. Microstructure of Myosin Gels

##### Examination by Confocal Laser Scanning Microscopy (CLSM)

**Figure 9:**
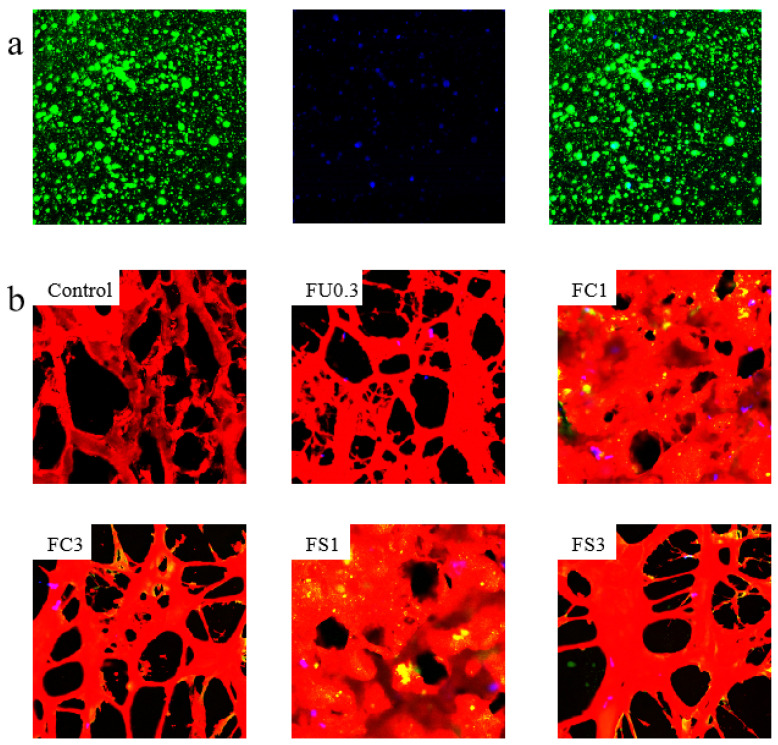
CLSM images of FU–vegetable oil emulsions (**a**) and myosin gel (**b**). Note: control: myosin gel; FU0.3: myosin-0.3%FU; FC1: myosin-0.3%FU-1% camellia oil; FC3: myosin-0.3%FU-3%camellia oil; FS1: myosin-0.3%FU-1% soybean oil; FS3: myosin-0.3%FU–3%soybean oil.

CLSM images can be used to reflect the distribution and aggregation of oil droplets in the gel matrix. The vegetable oil was dyed green with Nile red, and FU was dyed blue with Calcofluor White in Figure 9a. The result confirmed the formation of an O/W emulsion. In Figure 9b, the pink areas represent FU, the red areas represent protein networks, and the yellow areas represent oil droplets. The control group had large pores and formed loose microstructure; however, FU0.3 showed a more stable three-dimensional network structure. This result may be because the electrostatic interaction between the myosin and FU limited the movement of water molecules into the myosin gel [60]. Compared with the FU group, FC1 and FS1 showed a denser structure and smaller pores. This was attributed to the emulsion effect of FU on vegetable oil, which enhanced the cross-linking of protein–oil droplets. The above results were consistent with those of Wu et al. [61].

##### Examination by Light Microscopic Observation

**Figure 10:**
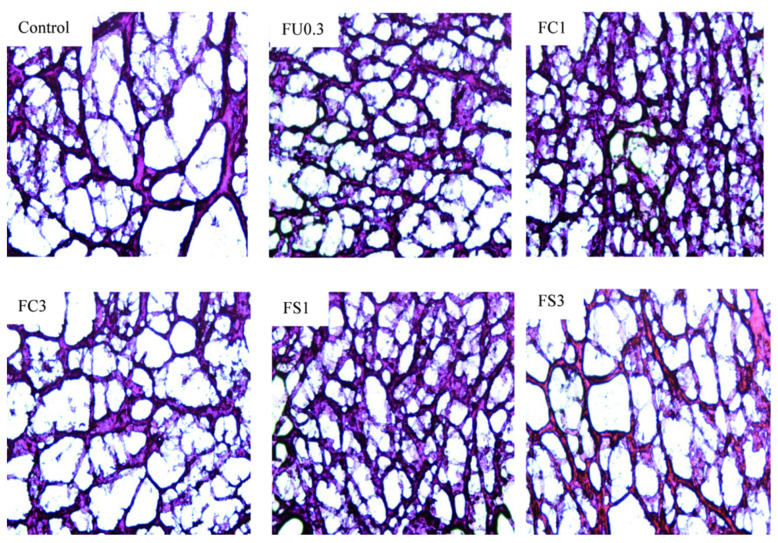
Changes in light microscope observation of the myosin gel. Note: control: myosin gel; FU0.3: myosin-0.3%FU; FC1: myosin-0.3%FU-1% camellia oil; FC3: myosin-0.3%FU-3%camellia oil; FS1: myosin-0.3%FU-1% soybean oil; FS3: myosin-0.3%FU-3%soybean oil.

As shown in Figure 10, the interconnections between the FU and proteins in continuous phase thus form a dense gel network. Moreover, FU was conducive to the formation of the emulsion droplets with smaller particle sizes and larger surface areas, which promoted the formation of a uniform gel network. However, when the oil content continued to increase, the cross-linking of the protein and density of the network structure of the gel decreased [62]. This was because vegetable oil increased the droplet size, thus affecting the continuity of the gel network and weakening the strength of the gel [63].

## 4. Conclusions

In this study, the FU–vegetable oil emulsion increased the particle size, turbidity, surface hydrophobicity, and total sulfhydryl content of myosin, which had a positive correlation with the content of vegetable oil. The interaction between the FU–vegetable oil and myosin changed the tertiary structures of myosin according to a multispectral analysis. The FU–vegetable oil emulsion promoted the unfolding, aggregation, and conformational transformation of myosin and formed more stable and finer oil droplets, thus reinforcing the gel network structure, wherein the 0.3% FU-1% vegetable oil group synergistically improved the performance of the myosin gel. FU–vegetable oil emulsions can effectively improve the quality of myosin gels, which provides a theoretical foundation for the processing of surimi products.

## Figures and Tables

**Table 1:** Effects of FU–vegetable oil emulsion on myosin gel TPA.

Sample	Hardness (g)	Springiness (%)	Cohesiveness (g)	Chewiness (g)
Control	343.88 ± 4.57 d	0.91 ± 0.03 b	0.62 ± 0.02 b	193.11 ± 4.03 c
FU0.3	387.41 ± 3.26 b	0.92 ± 0.01 b	0.65 ± 0.01 ab	230.42 ± 3.67 b
FC1	406.80 ± 5.90 a	0.94 ± 0.01 a	0.66 ± 0.01 a	252.75 ± 8.45 a
FC3	364.30 ± 2.29 c	0.91 ± 0.01 b	0.62 ± 0.01 b	213.53 ± 4.17 b
FS1	399.33 ± 9.66 ab	0.93 ± 0.01 ab	0.65 ± 0.02 ab	249.72 ± 4.12 a
FS3	368.02 ± 3.22 c	0.91 ± 0.02 b	0.62 ± 0.01 b	212.87 ± 5.91 b

Note: control: myosin gel; FU0.3: myosin-0.3%FU; FC1: myosin-0.3%FU-1% camellia oil; FC3: myosin-0.3%FU-3%camellia oil; FS1: myosin-0.3%FU-1% soybean oil; FS3: myosin-0.3%FU-3%soybean oil. Different lowercase letters (a–d) in the same column indicate significant differences at *p* < 0.05.

**Table 2:** Effects of the FU–vegetable oil emulsion on myosin gel color and WHC.

Sample	L*	a*	b*	Whiteness	WHC (%)
Control	84.05 ± 0.57 a	−1.86 ± 0.04 d	−4.14 ± 0.49 c	83.40 ± 0.63 ab	44.82 ± 3.87 b
FU0.3	83.21 ± 0.71 b	−1.55 ± 0.26 bc	−2.64 ± 0.80 ab	82.91 ± 0.72 b	52.90 ± 2.69 a
FC1	83.54 ± 0.11 ab	−1.32 ± 0.29 a	−2.94 ± 0.38 ab	83.22 ± 0.05 ab	53.46 ± 2.68 a
FC3	83.99 ± 0.68 a	−1.56 ± 0.12 bc	−2.24 ± 0.51 a	83.75 ± 0.70 a	47.78 ± 3.91 b
FS1	83.76 ± 0.11 ab	−1.49 ± 0.01 b	−3.1 ± 0.24 b	83.40 ± 0.12 ab	53.31 ± 1.93 a
FS3	84.02 ± 0.22 a	−1.69 ± 0.12 c	−2.68 ± 0.39 ab	83.70 ± 0.70 a	48.83 ± 3.52 ab

Note: control: myosin gel; FU0.3: myosin-0.3%FU; FC1: myosin-0.3%FU-1% camellia oil; FC3: myosin-0.3%FU-3%camellia oil; FS1: myosin-0.3%FU-1% soybean oil; FS3: myosin-0.3%FU-3%soybean oil. Different lowercase letters (a–d) in the same column indicate significant differences at *p* < 0.05.

## Data Availability

The original contributions presented in this study are included in this article; further inquiries can be directed to the corresponding author.
